# Targeting ferroptosis by trimetazidine and probiotics to attenuate 5-fluorouracil-induced intestinal mucositis in mice

**DOI:** 10.1007/s00210-025-04936-9

**Published:** 2026-01-16

**Authors:** Lamiaa M. El Ballat, Alshimaa Aboalsoud, Rasha Osama Elesawy, Fleur F. Abd Elmonem, Sabeha E. Hedya

**Affiliations:** Pharmacology Department, Faculty of Medicine, Tanta University, Tanta, Egypt

**Keywords:** 5-Fluorouracil, Trimetazidine, Probiotics, Mucositis, Ferroptosis, Nrf2 activation

## Abstract

Intestinal mucositis is a common side effect in cancer patients receiving chemotherapy, yet effective treatment options are lacking. This study aimed to investigate the potential protective effects of trimetazidine and probiotics combination in a 5-FU-induced intestinal mucositis model. We hypothesized that these agents would attenuate mucositis through anti-inflammatory and antioxidant effects associated with modulation of ferroptosis-related pathways, particularly through Nrf2-associated regulatory mechanisms. 5-fluorouracil in a dose of 50 mg/kg/day i.p. for 6 consecutive days starting from the 11th to the 16th day for induction of chemotherapy-induced intestinal mucositis (CIM) and mice were divided into five equal groups as follows: control group, untreated group, trimetazidine group, probiotics group, and trimetazidine + probiotics group. The effects of these drugs on inflammation, oxidative stress, and ferroptosis were assessed. Treatment with trimetazidine and probiotics in combination produced further improvement in disease activity, reflected by increased body weight and reduced diarrhea scores. Significant reductions were observed in IL-1β and MDA levels, with parallel increases in SOD, GPx4, CAT, Nrf2, and SLC7A11. The combination also provided additional improvement in intestinal structure (villus height, crypt depth, and enteritis score) compared with each monotherapy group. Either trimetazidine or probiotics alleviated 5-FU-induced intestinal mucositis and might be considered promising candidates for preventing CIM by anti-inflammatory, antioxidant, and mechanisms associated with modulation of ferroptosis-related pathways.

**Supplementary Information:**

The online version contains supplementary material available at 10.1007/s00210-025-04936-9.

## Introduction

Multiple beneficial anti-tumor effects are linked to cancer chemotherapy; nevertheless, there are also significant side effects and long-term safety concerns for cancer patients receiving chemotherapy (Sougiannis et al. 2021). Chemotherapeutics-induced intestinal mucositis (CIM) is one of the most frequent off-target toxicities. Between 40 and 100% of cancer patients are affected (Sonis et al. 2015; Villa and Sonis 2015; Dahlgren et al. 2021).

Pathophysiology of mucositis involves a variety of mechanisms that extend beyond the immediate damage to the epithelium caused by chemotherapy. Reactive oxygen species (ROS) generation is the main inducer that initiates mucosal damage after chemotherapy and causes tissue inflammation and mucositis in many ways (Ribeiro et al. 2016).

Chemotherapy activates the multiprotein complex inflammasome, which increases the creation of ROS that activates inflammatory mediators, which then trigger caspase activity and the activation of IL-1β and IL-18. The well-known NFκB activators, IL-1β and IL-18, suggest that ROS-driven NFκB activation might be partially reliant on inflammasome formation. Thus, activation of the inflammasome appears to be crucial for chemotherapy-induced mucositis (Amaral et al. 2012).

Despite extensive work on oxidative stress and inflammation in CIM, ferroptosis-targeted therapeutic strategies remain largely unexplored, and no studies to date have evaluated pharmacologic agents capable of modulating ferroptosis in this condition.

Ferroptosis occurs when oxidative stress causes excessive damage to membrane lipids. This iron-dependent process represents a unique type of regulated cell death, different from apoptosis (Jenkins et al. 2020).

Solute carrier family 7 member 11 (SLC7A11) and solute carrier family 3 member 2 (SLC3A2) are the two subunits that make up System Xc (cystine/glutamate antiporter). Dysfunction of the Xc-dependent antioxidant defense mechanism leads to oxidative damage, which in turn triggers ferroptosis (Tu et al. 2021).

It has been shown that maintaining glutathione (GSH) synthesis or increasing system Xc^−^ or GPx4 activity protects cells under oxidative stress (Jiang et al. 2021).

A group of cytoprotective genes is regulated by the redox-sensitive transcription factor, nuclear factor erythroid-related factor 2 (Nrf2). Emerging data suggest that Nrf2 controls SLC7A11 and GPx4, which are two important targets of ferroptosis (Dodson et al. 2019). Nrf2 also regulates catalase (CAT), superoxide dismutase (SOD), glutathione S-transferase, and exerts anti-inflammatory effects (He et al. 2020). Given this central role of Nrf2 in oxidative, inflammatory, and ferroptosis-related pathways implicated in CIM, agents capable of activating Nrf2 are increasingly recognized as promising candidates for mitigating CIM.

For patients suffering from CIM, the only effective strategy remains dose reduction of cytotoxic chemotherapy, but increasing understanding of CIM pathophysiology opens new therapeutic avenues (Dahlgren et al. 2021).

5-Fluorouracil (5-FU) is widely used, yet ~80% of treated patients develop gastrointestinal mucositis (Chang et al. 2012). The mouse 5-FU model is simple, low-cost, and highly representative of human disease (Vodenkova et al. 2020; Huang et al. 2022).

Trimetazidine (TMZ) is a cytoprotective agent with well-established antioxidant and anti-inflammatory actions. It has been shown to enhance endogenous antioxidant defenses in part through Nrf2-associated pathways, providing a clear mechanistic rationale for investigating its role in conditions characterized by oxidative stress and ferroptosis-related injury, such as chemotherapy-induced mucositis. In addition, accumulating evidence indicates that TMZ can mitigate several chemotherapy-related toxicities through its ability to reduce oxidative damage, suppress inflammatory responses, and modulate pathways linked to ferroptosis (Lopaschuk et al. 2003; Ateyya et al. 2016).

Probiotics were defined by FAO/WHO Expert Consultation as live microorganisms that boost the host’s health when given in sufficient quantities (Meurman and Stamatova 2007).

Probiotics (*Lactobacillus*, *Bifidobacterium* species) reduced 5-FU–induced mucositis in several models, offering anti-inflammatory and antioxidant benefits (Fang et al. 2014). Therefore, they could be used in conjunction with chemotherapy to promote the reduction of CIM, which is promising for enhancing the quality of life of patients receiving chemotherapy (Mi et al. 2017; Chang et al. 2018).

Importantly, TMZ and probiotics exhibit complementary mechanisms: TMZ targeting metabolic and redox pathways, including Nrf2 activation, and probiotics modulating inflammation, oxidative stress, and gut barrier integrity, providing a clear rationale for testing their combined effects.

The present study aimed to evaluate the effects of trimetazidine, alone or in combination with probiotics, on inflammation, oxidative stress, and ferroptosis-related pathways in 5-FU–induced intestinal mucositis in mice, with particular focus on the Nrf2/GPx4/SLC7A11 antioxidant axis. Accordingly, we hypothesized that these interventions would attenuate mucositis through improvements in redox balance and inflammatory status, and the study objectives were formulated to evaluate these effects and clarify their potential links to Nrf2 signaling.

## Materials and methods

### Ethical consideration

All experimental methods were conducted in the Medical Pharmacology Department of the Faculty of Medicine, Tanta University, Egypt. The handling of animals and all experimental procedures were adopted by the institutional “Research Ethics Committee, REC”, Faculty of Medicine, Tanta University, Egypt (Approval no.36264 MD 15/1/23).

### Drugs and chemicals

5-FU ampoules (250 mg/5 ml), a product of AMRIYA Pharmaceutical Industries (Egypt), was diluted in saline to a final concentration of 12.5 mg/ml and administered by intraperitoneal injection (i.p.) in a dose of 50 mg/kg/day for 6 consecutive days. Trimetazidine white powder, purchased from Maps Laboratories PVT. LTD. (India), was dissolved in distilled water to a final concentration of 2.5 mg/ml and administered by oral gavage in a dose of 20 mg/kg/day. *Bifidobacterium bifidum* and *Lactobacillus acidophilus* capsules, a product of SIT Laboratorio farmaceutico (Mede, Italy), were diluted in saline to a final concentration of 5 × 10^7^ CFU/ml and administered by oral gavage in a dose of 1 × 10^7^ CFU/mice/day. The selected strains (*B. bifidum* and *L. acidophilus*) were chosen based on prior evidence supporting their anti-inflammatory and antioxidant activity in mucositis and intestinal injury models (Kato et al. 2017).

These doses fall within the effective and widely used ranges in murine models, where TMZ at 20 mg/kg has repeatedly demonstrated antioxidant and anti-inflammatory efficacy, and probiotic doses between 10^6^ and 10^9^ CFU/mouse/day are considered adequate to induce functional gastrointestinal effects (Jain et al. 2010; Yeung et al. 2015).

Other chemicals supplied by Adwic, El-Nasr Pharmaceutical Chemical Co., EGYPT, were of analytical grades.

### Experimental animals

This study was carried out on 50 healthy Swiss Albino mice, 8–12 weeks old and weighing around 20 to 30 g. Mice were housed in an animal laboratory room in wire mesh cages under strict hygienic measures and had free access to a standard animal diet and water ad libitum. The animals were allowed to acclimatize for 2 weeks under a 12/12 h dark/light cycle, and under controlled environmental conditions, including a temperature of 22 ± 2 °C, relative humidity of 50–60%, and adequate ventilation. Each experimental group included 10 mice (*n* = 10), and all animals were included in all biochemical, clinical, and histological analyses, with no exclusions or mortality”.

### Study design

**Fig. 1:**
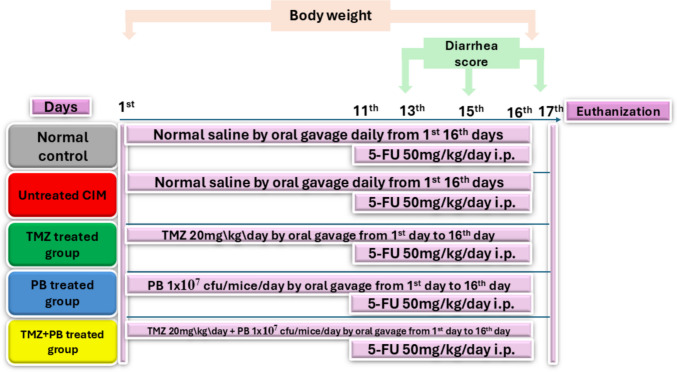
A schematic representation of experimental study design. 5-fluorouracil (5-FU) was administered intraperitoneally at 50 mg/kg/day on days 11–16 to induce intestinal mucositis. Trimetazidine (TMZ, 20 mg/kg/day p.o.) and probiotics (PB, 1 × 10⁷ CFU/mouse/day p.o.) were administered daily from days 1 to 16 according to group allocation. The five experimental groups were control, untreated, TMZ, PB, and TMZ + PB. Tissue collection was performed on day 17

Mice were divided randomly into five equal groups (*N* = 10). Group 1 served as control and received saline by i.p injection from the 11th to the 16th day and per os (p.o.) from 1 to 16 days. CIM was induced in mice of Groups 2, 3, 4 and 5 using 5-FU in a dose of 50 mg/kg/day by i.p for 6 consecutive days starting from the 11th to the 16th day (Hamouda et al. 2017). Group 2 served as untreated CIM and received normal saline p.o. daily. Mice in Group 3 were treated with trimetazidine (TMZ) in a dose of 20 mg/kg/day p.o. (Zhang et al. 2020). Mice in Group 4 were treated with probiotics (PB) in a dose of 10^7^ CFU daily p.o. (Yeung et al. 2020). Mice in Group 5 were treated with TMZ + PB combination in the same dosage regimen mentioned before. The treatment protocol for Groups 3, 4, and 5 was started from the 1 st to the 16th day (Fig. 1)

### Change in body weight (B.W.)

The body weight (B.W.) of each mouse was recorded at the start point of the experiment (1^st^ day) and the endpoint of the experiment (17^th^ day) to calculate the percent (%) change in B.W. according to the following formula:

$$\frac{Final\;B.W\;-\;Initial\;B.W}{\;Initial\;B.W}\times100$$

### Diarrhea score

The diarrhea score was done on 13th, 15th, and 17th days based on Bowen’s score system and classified into four grades according to the stool consistency: 0, normal stool; 1, slightly wet and soft stool indicating mild diarrhea; 2, wet and unformed stool indicating moderate diarrhea; and 3, watery stool indicating severe diarrhea (Bowen et al. 2007).

### Intestinal specimen collection & processing

At the endpoint of the experiment, mice were euthanized under anesthesia using phenobarbital sodium in a dose of 50 mg/kg i.p. (Laferriere and Pang 2020). A segment of 3–5 cm was excised from the jejunum and colon and rinsed with 0.9% normal saline and then divided into two parts, where one part was fixed in 10% buffered neutral formalin for 24 h. The other parts of the jejunum and mid colon from the respective group were washed, weighed, and homogenized in 5 ml cold phosphate-buffered saline (pH 7.4) per gram of tissue using a digital ultrasonic homogenizer. The homogenate was centrifuged at 4000 rpm for 15 min at 4°C.

### Histopathological examinations

The formalin-fixed jejunal and colon tissues were dehydrated in ascending series of ethanol concentrations and embedded in paraffin wax. Sections of 4 µm thicknesses were cut vertically using a Leica microtome and fixed on a glass slide for H&E and Prussian blue stain for examination of the histopathological changes and iron deposition, respectively, by light microscope (Leica DM 2000, Leica microscopy and scientific instruments group, Germany). Villi heights (from the villus tip to the villus-crypt junction) and crypt depths (invagination depth between adjacent villi) were calculated by measuring twenty well-oriented villi and crypts. The lesion score of jejunum was done by two independent pathologists who were blinded toward group characteristics and outcomes and assessed by using a modified histopathological score system and graded as follows: Score 0, normal histological findings; Score 1, mucosa: loss of crypt architecture, sparse inflammatory cell infiltration, vacuolization, and edema; muscle layer: normal. Score 2, mucosa: crypt necrosis, intense inflammatory cell infiltration, vacuolization, and edema; muscle layer: normal. Score 3, mucosa: crypt necrosis, intense inflammatory cell infiltration, vacuolization, and edema; muscle layer: edema, vacuolization, and neutrophilic infiltration (MacPherson and Pfeiffer 1978; Costa et al. 2019).

### Assay of oxidative stress status

The resultant fractions from the jejunal and colon homogenate centrifugation were used for assay of SOD (µ/g tissue), Gpx4 (µ/g tissue), CAT (µ/g tissue), MDA (nmol/g tissue) using spectrophotometry kits supplied by Biodiagnostic Company; Giza, Egypt (Cat. No SD 25 21, GP 2524, CA 25 17, and MD 2529); respectively.

Assay of inflammatory status and ferroptosis by enzyme-linked immunosorbent assay (ELISA) of IL-1β, SLC7A11, and Nrf2 levels

The other parts of supernatants from the jejunal and colon were analyzed for IL-1β (pg/ml), SLC7A11 (ng/l), and Nrf2 (ng/dl) levels by ELISA kits supplied by Sun Red Biotechnology Company, Shanghai, China (Catalogue No. 201-02−0193, 201-02−1684, and 201-02−1559; respectively) according to the manufacturer’s instructions.

## Statistical analysis

All obtained data were tabulated and statistically analyzed using GraphPad Prism, version 9.5.1 for Windows, USA. Shapiro–Wilk test was performed for assessment of normality. The parametric values were expressed as mean ± standard deviation (SD) and compared by One Way ANOVA followed by post-hoc Tukey’s tests, and the paired *t*-test. The non-parametric values were expressed as median (Interquartile range, IQR) and compared by Kruskal–Wallis and Mann–Whitney *U* tests. Biological replicates were defined as individual animals, and no data was excluded from the analysis.

The significance was considered at values of *p* < 0.05.

## Results

### Effect of the different treatments on body weight change

**Fig. 2:**
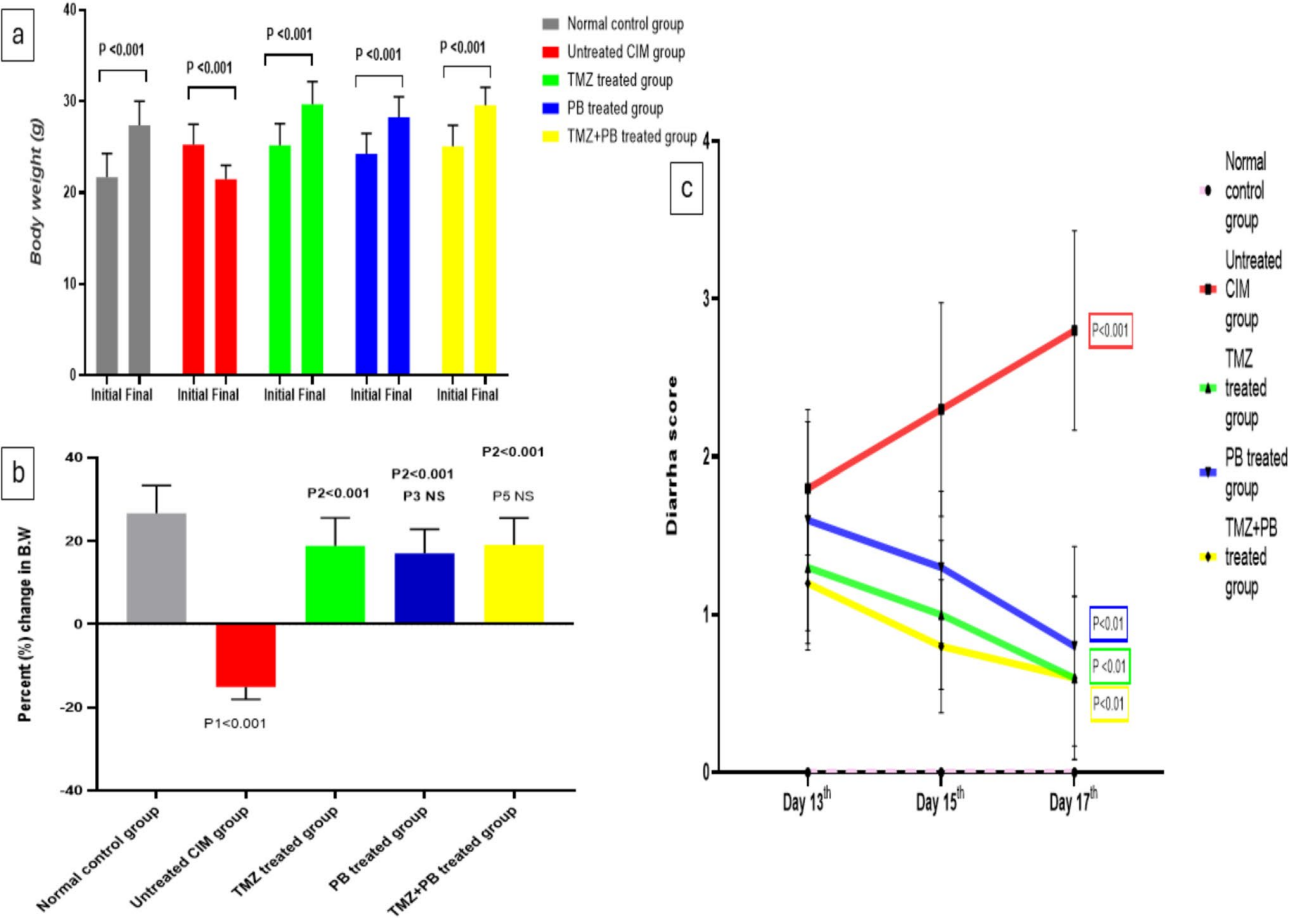
**a** Differences between initial and final body weight (B.W.) within each experimental group. Data are presented as mean ± SD (*n* = 10 per group); NS, non-significant. (P): final B.W. vs. initial B.W. within the same group (paired *t*-test). **b** Effect of the different treatments on the percentage change in B.W. (P1): untreated CIM vs. normal control; (P2): TMZ, PB, and TMZ + PB vs. untreated CIM; (P3): PB group 4 vs. TMZ group 3); P4, TMZ + PB vs. TMZ; (P5) TMZ + PB vs. PB. One-way ANOVA followed by Tukey’s post hoc test. **c** Diarrhea score at different time points within each group (13th, 15th, and 17th days). Repeated measures non-parametric analysis was performed using the Friedman test, followed by Dunn’s post-hoc test for pairwise comparisons. Data are expressed as median (IQR). (P): day 17th vs day 13th

The administration of 5-FU resulted in a marked reduction in body weight, reflected by a negative percent change of −15.07% ± 3.01% compared with the normal control group (+ 26.75% ± 6.66%). Treatment with TMZ, PB, or their combination substantially attenuated this loss, producing positive percent changes of +18.86% ± 6.73%, +17.11% ± 5.75%, and +19.14% ± 6.39%, respectively. These values indicate that all three treatments markedly improved body-weight outcomes relative to the untreated CIM group (*p* < 0.001) (Fig. 2a and b and Supplementary Table 1)

### Effect of the different treatments on diarrhea score

The administration of 5-FU showed a significant increase in diarrhea score on the 17th day as compared to the 13th day. While the administration of TMZ and/or PB each alone or in combination showed a significant decrease in diarrhea score on the 17th day as compared to the 13th day **(**Fig. 2c).

### Effects of different treatments on oxidative stress markers in the intestinal tissues

**Fig. 3:**
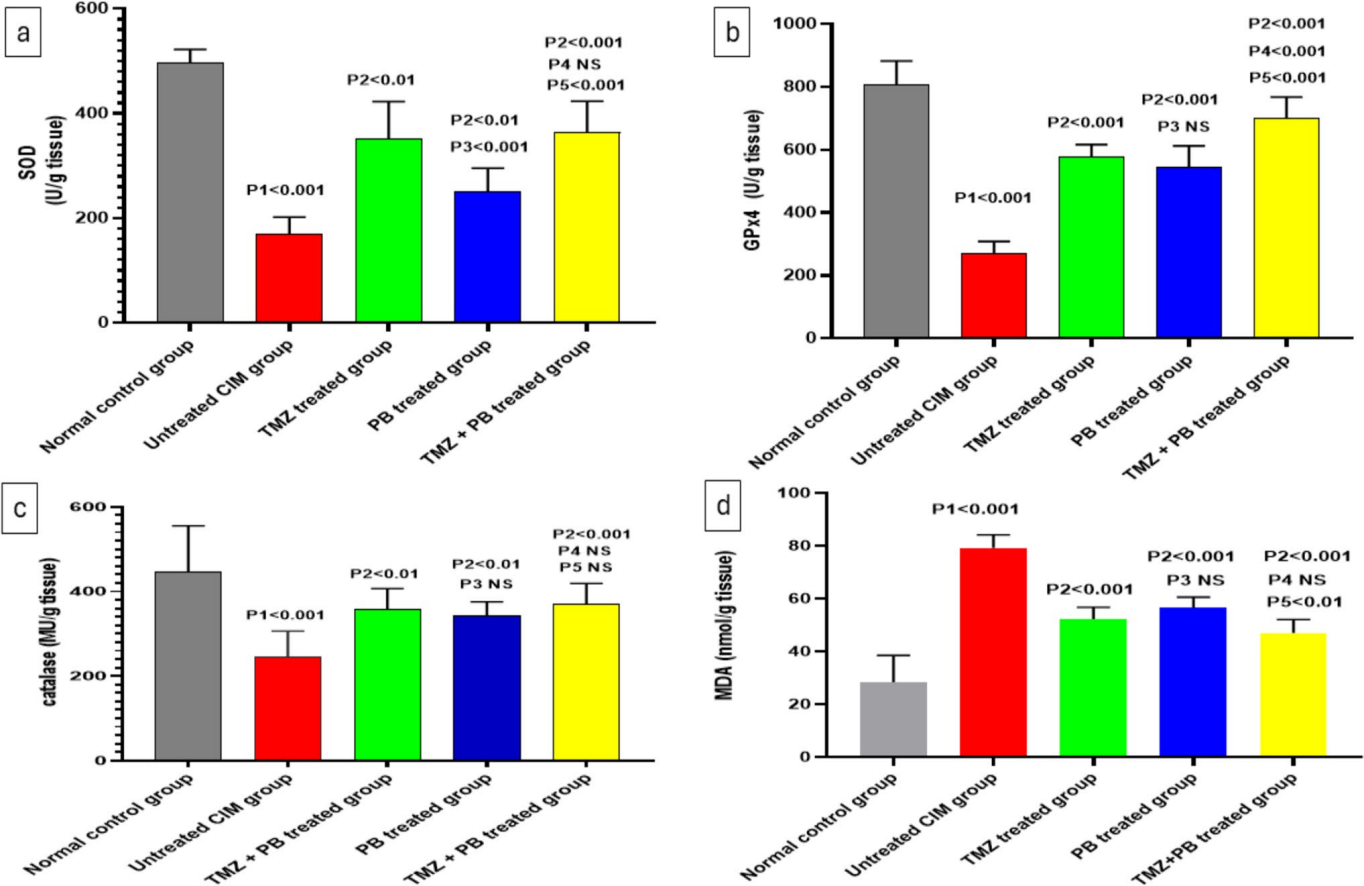
Effect of the different treatments on **a** SOD level, **b** GPx4 level, **c** CAT level, and **d** MDA level in intestinal tissue in the studied groups. Data are presented as mean ± SD (*n* = 10 per group) NS, non-significant. Statistical analysis was performed using one-way ANOVA followed by Tukey’s post hoc test. (P1): untreated CIM vs normal control; (P2): TMZ, PB, and TMZ + PB vs untreated CIM; (P3): Group 4 PB vs Group 3 TMZ; (P4): TMZ + PB vs TMZ; (P5): TMZ + PB vs PB

There was a significant decrease in SOD, Gpx4, and CAT tissue levels and a significant increase in MDA tissue level in the untreated CIM group as compared to the normal control group. The administration of TMZ or PB alone or as a combination resulted in a significant increase in SOD, Gpx4, and CAT tissue levels and a significant decrease in MDA-level, as compared to the untreated CIM group. The TMZ + PB combination produced the strongest biochemical response. It showed GPx4 levels than both TMZ alone and PB alone, Lower MDA levels compared with PB alone. Additionally, the TMZ group had higher SOD activity than the PB group (Fig. 3a, b, c, and d).

### Effects of different treatments on IL-1β levels in the intestinal tissues

**Fig. 4:**
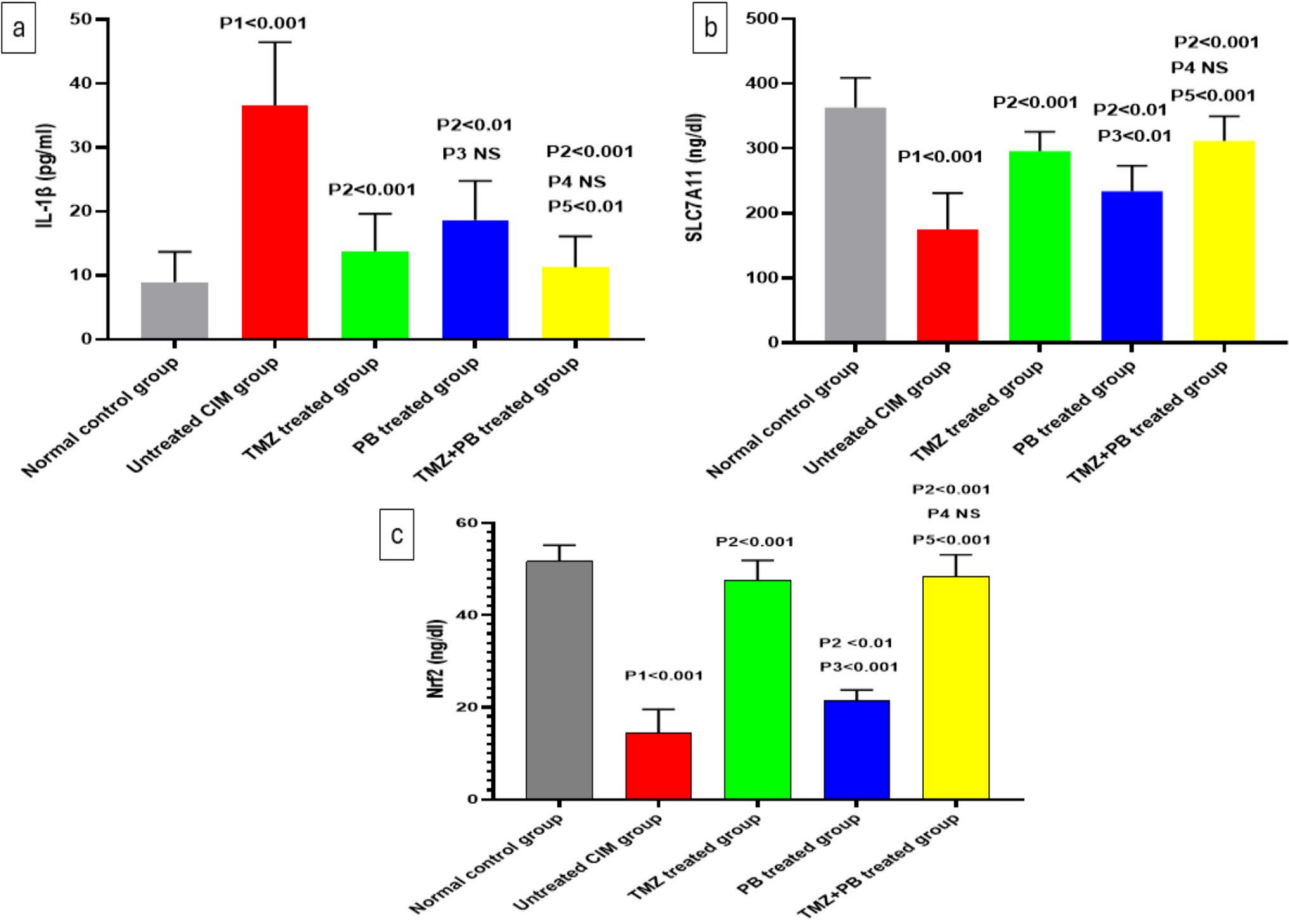
Effect of the different treatments on **a** IL-1β level, **b** SLC7A11 level, and **c** Nrf2 level in intestinal tissue in the studied groups. Data are presented as mean ± SD (*n* = 10 per group); NS, non-significant. Statistical analysis was performed using one-way ANOVA followed by Tukey’s post hoc test. (P1): untreated CIM vs normal control; (P2): TMZ, PB, and TMZ + PB vs untreated CIM; (P3): Group 4 PB vs Group 3 TMZ; (P4): TMZ + PB vs TMZ; (P5): TMZ + PB vs PB

The inflammatory marker IL-1β increased significantly in the untreated CIM group when compared to the control group. Treatment with TMZ and PB each alone or in combination resulted in a significant decrease in IL-1β tissue level as compared to the untreated CIM group. The decrease was more prominent in the combination group and was significant when compared to the use of PB alone (Fig. 4a).

### Effects of different treatments on SLC7A11 levels and Nrf2 levels in the intestinal tissues

SLC7A11 is a key component of system Xc^−^ and a critical regulator of glutathione-dependent antioxidant defense, making it an essential indicator of ferroptosis-related vulnerability in intestinal mucositis. There was a significant decrease in SLC7A11 and Nrf2 tissue levels in the 5-FU-induced intestinal mucositis group as compared to the control group. Treatment with TMZ and PB each alone or in combination resulted in a significant increase in SLC7A11 and Nrf2 tissue levels as compared to the untreated CIM group. These results were more significant in the TMZ group and TMZ + PB combination group, as compared to the use of the PB drug alone (Fig. 4b and c).

### Histopathological examination of stained sections (H&E), villi height, crypts depth, and enteritis score of the jejunum of different studied groups

**Fig. 5:**
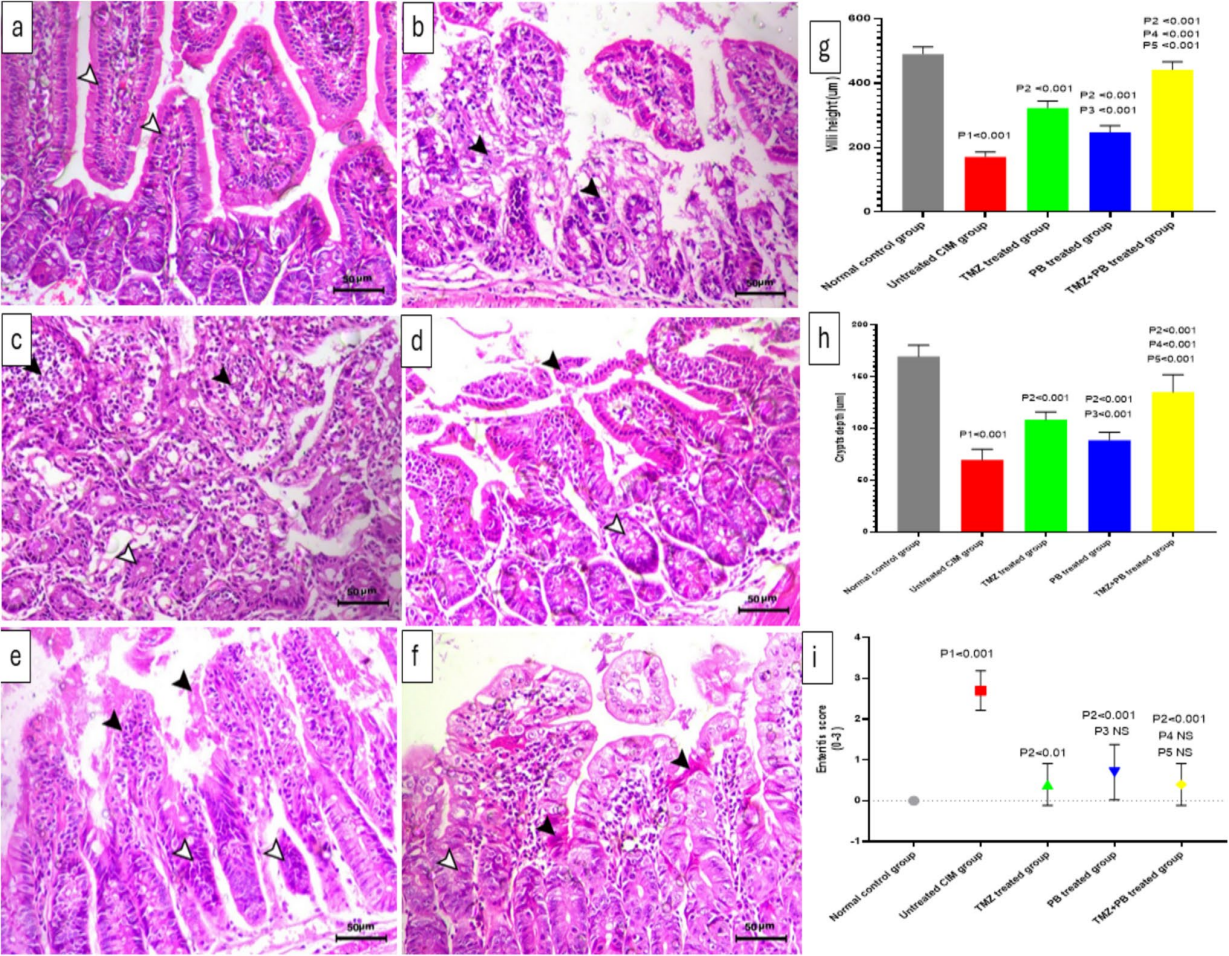
H&E stained sections from of the jejunum of **a** normal control group showing normal intestinal villi and crypts with normal pseudostratified epithelium (white arrowheads); H&E, × 200; scale bar = 50 µm. **b** and **c** Untreated CIM group showing severe degree of necrotic enteritis associated with marked necrosis of the intestinal villi, mononuclear inflammatory cell infiltration (black arrowheads), and degenerative changes within the basal intestinal crypts (white arrowhead). **d** Trimetazidine-treated group showing mild necrotic and desquamative changes of the covering epithelium (black arrowhead) and mild degenerative changes within the intestinal crypts (white arrowhead). **e** Probiotics-treated group showing decrease the necrotic lesions which mostly seen within the covering epithelium of the intestinal villi (black arrowheads) and with degenerative changes of the lining epithelium of the intestinal crypts associated with nuclear pyknosis (white arrowheads). **f** Trimetazidine and probiotics combination-treated group showing normal intestinal mucosa with normal covering and lining epithelium with only mild apoptotic changes of some epithelial cells (black arrowheads) and with marked regeneration of the intestinal crypts (white arrowhead); H&E, × 200; scale bar = 50 µm. **g** Villi height (µm) and **h** crypts depth (µm); values are expressed as mean ± SD (*n* = 10 per group) NS, non-significant. Data were analyzed using one-way ANOVA followed by Tukey’s post hoc test; **i** enteritis score. Values are expressed as median (IQR) and were analyzed using the Kruskal–Wallis test followed by Mann–Whitney *U* tests. (**P1)**: untreated CIM vs normal control; (P2): TMZ, PB, and TMZ + PB vs untreated CIM; (P3): Group 4 PB vs Group 3 TMZ; (P4): TMZ + PB vs TMZ; (P5): TMZ + PB vs PB

The control group displayed normal intestinal architecture with well-preserved villi, normal crypt structure, and an absence of enteritis (Fig. 5a and i). In contrast, 5-FU administration caused marked mucosal injury, evident by severe necrotic enteritis, villous destruction, crypt degeneration, and dense mononuclear inflammatory infiltration, together with a significant reduction in villi height and crypt depth and a significant increase in enteritis score compared with the control group (Fig. 5b, c, and i).

Treatment with either TMZ or PB clearly ameliorated the 5-FU–induced histopathological damage, showing noticeable restoration of villous structure, reduced epithelial necrosis, and improved crypt morphology. These improvements were accompanied by significant increases in villi height and crypt depth relative to the untreated CIM group (Fig. 5d and e).

The TMZ + PB combination produced the most pronounced histological recovery, with nearly normal mucosal architecture and only mild residual apoptotic epithelial changes (Fig. 5f). This group also showed significantly higher villi height and crypt depth (Fig. 5g and h) compared with both the untreated CIM group and each monotherapy. Consistently, TMZ, PB, and their combination all resulted in a significant reduction in enteritis score compared with the untreated CIM group (Fig. 5i).

### Histopathological examination of colon of different studied groups

**Fig. 6:**
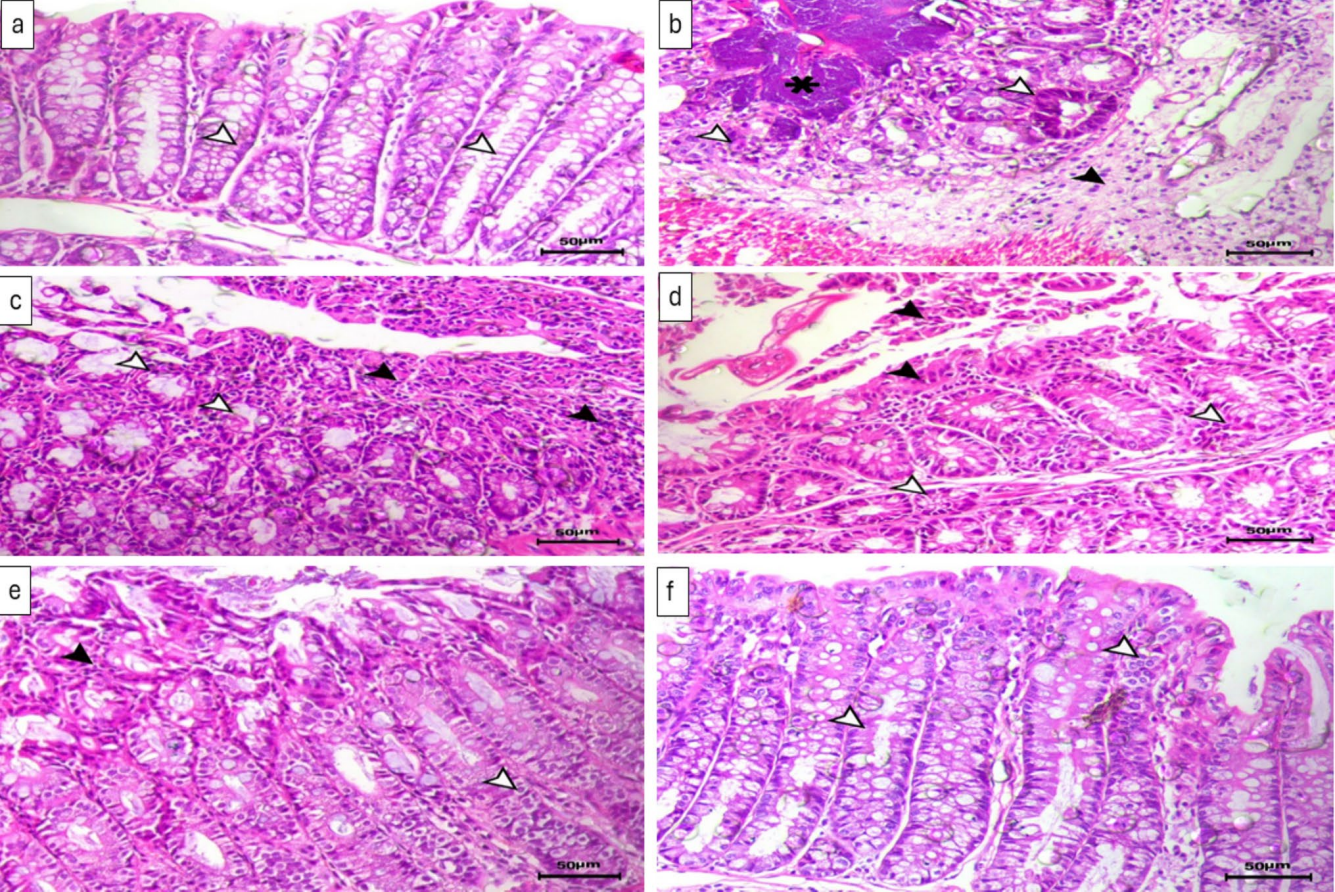
H&E stained sections from colon tissues of **a** normal control group showing normal mucosa with normal intestinal crypts containing normal goblet cells (arrowheads). **b** and **c** Untreated CIM group showing severe degree of necrotic colitis associated with luminal necrotic core (asterisk), necrosis of the intestinal glands (white arrowheads) and marked mucosal and submucosal edema associated with mononuclear inflammatory cells infiltration (black arrowhead). **d** Trimetazidine-treated group showing decreased necrotic lesions, mild desquamation of the lining mucosal layers (black arrowheads), and degenerative changes within the basal intestinal glands (white arrowheads). **e** Probiotics-treated group showing focal desquamation of the covering epithelium (black arrowhead) and vacuolar degenerative changes within the lining epithelium of the intestinal crypts (white arrowhead). **f** Trimetazidine and probiotics combination-treated group showing marked improvement in both degeneration and necrosis of the intestinal crypts (arrowheads indicates normal epithelial lining of the crypts); H&E, × 200; bar = 50 µm

The control group showed intact mucosa with normal intestinal crypts containing normal goblet cells (Fig. 6a). The administration of 5-FU resulted in necrotic enteritis in the form of necrosis of the apical portion of the intestinal mucosa and degeneration of the basal intestinal glands (Fig. 6b and c). TMZ (Fig. 6d) or PB (Fig. 6e), either alone or together (Fig. 6f), resulted in improvements in the degenerative and necrotic changes of the intestinal crypts.

### Results of Prussian blue staining of jejunal and colon tissues

**Fig. 7:**
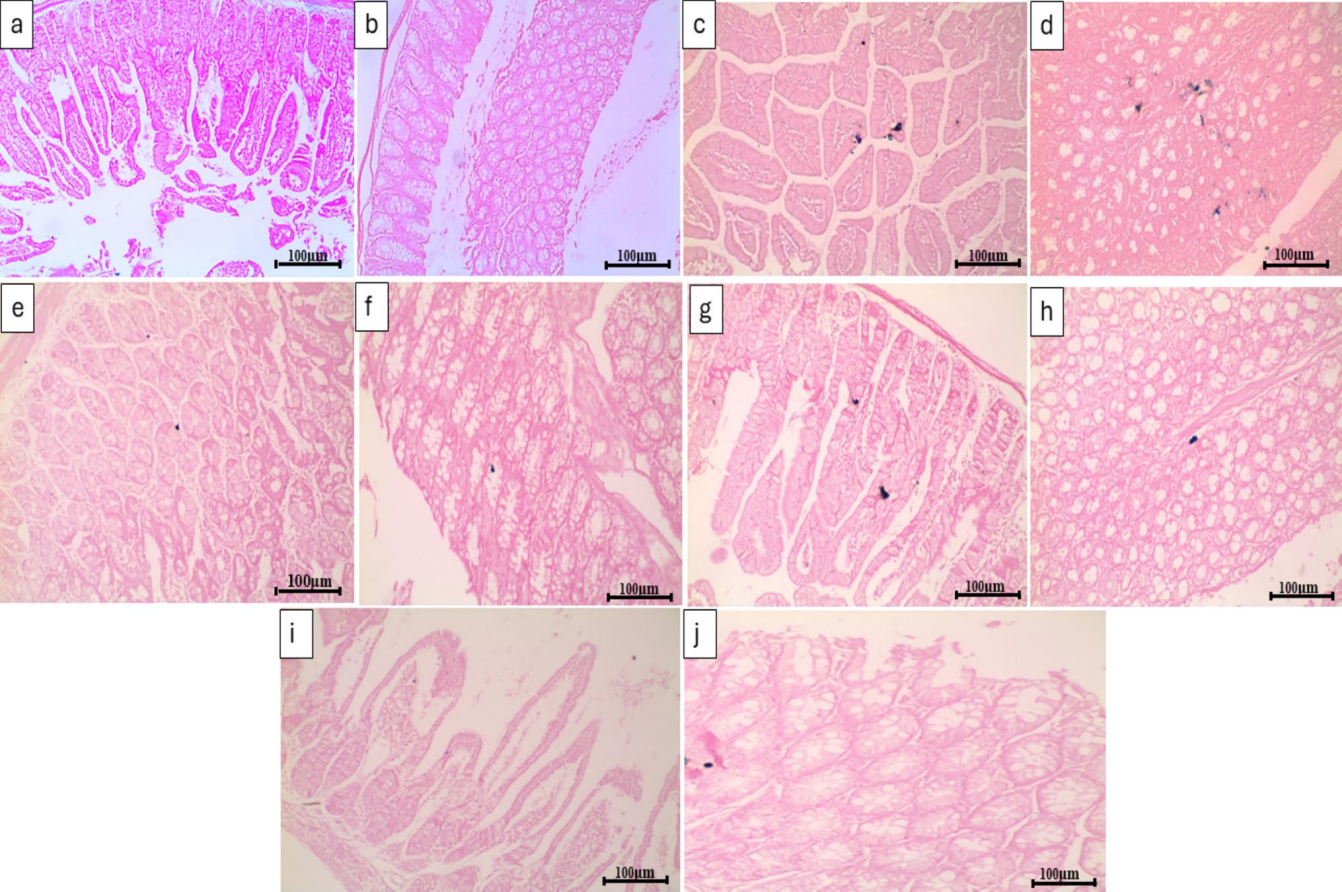
Histopathological examination by Prussian blue stain sections of **a** jejunum of normal control group showing no iron deposition, **b** colon of normal control group showing no iron deposition (negative Prussian blue staining), **c** jejunum of untreated CIM group showing severe iron deposition, **d** colon of untreated CIM group showing severe iron deposition, **e** jejunum of TMZ-treated group showing mild iron deposition, **f** colon of TMZ-treated group showing mild iron deposition, **g** jejunum of PB-treated group showing moderate iron deposition, **h** colon of PB-treated group showing moderate iron deposition, **i** jejunum of TMZ + PB-treated group showing mild iron deposition, and **j** colon of TMZ + PB-treated group showing mild iron deposition. All images × 200; bar = 100 µm

The normal control group showed a negative Prussian blue stain (Fig. 7a and b). Administration of 5-FU resulted in severe iron deposition in small and large intestinal stained tissues (Fig. 7c and d). TMZ alone or in combination with PB showed mild iron deposition (Fig. 7e, f, i, and j), while treatment with PB alone resulted in moderate iron deposition (Fig. 7g and h).

### Correlation of the diarrhea score with the tissue levels of SOD, GPx, CAT, MDA, IL-1β, SLC7A11, and Nrf2 in the untreated CIM group (group 2) (Fig. 8a, b, c, d, e, f, and g)

There was a significant negative correlation between diarrhea score and tissue levels of SOD, GPx, CAT, SLC7A11, and Nrf2; also, there was a significant positive correlation between diarrhea score and tissue levels of MDA and IL-1β.

**Fig. 8:**
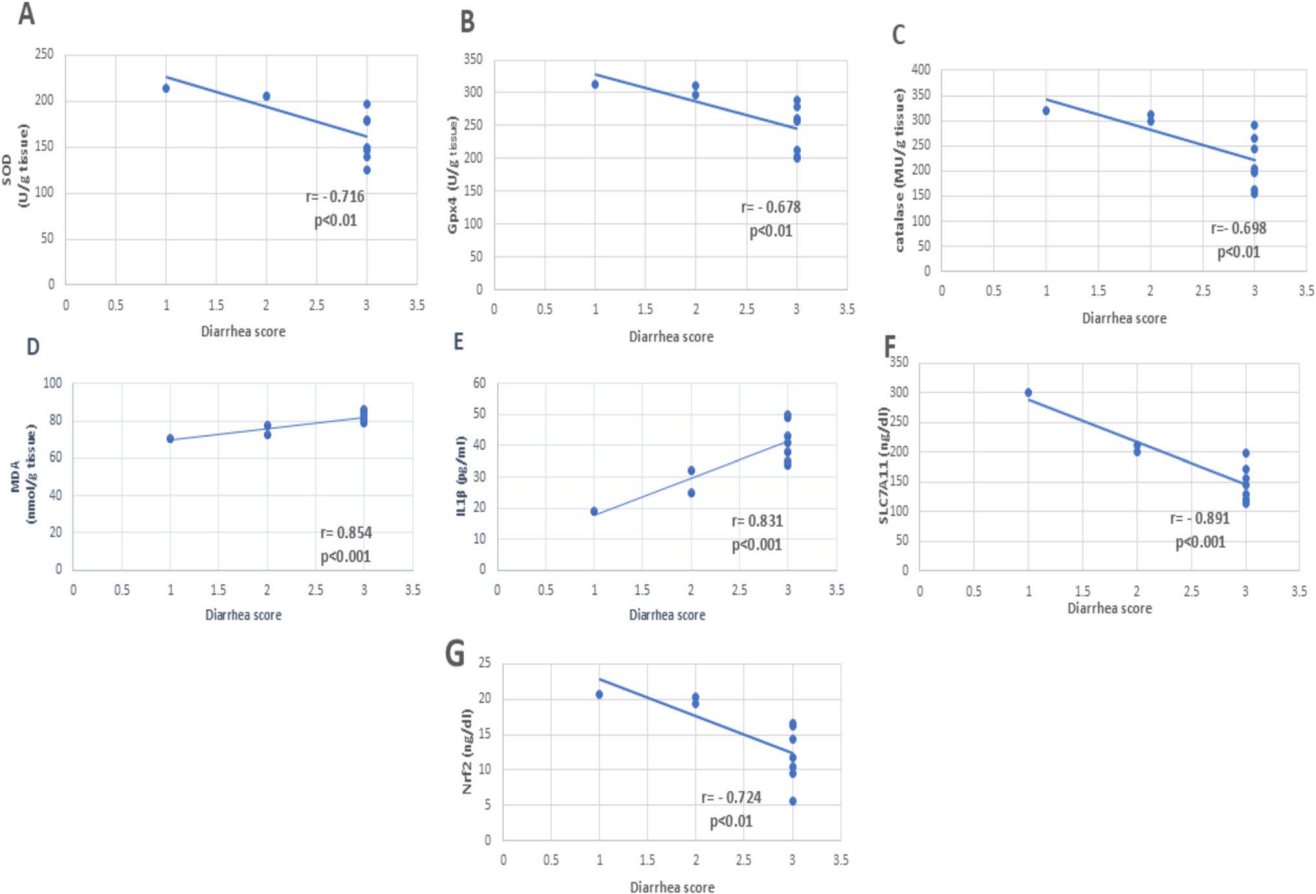
Correlation of diarrhea score with **A** superoxide dismutase (SOD), **B** glutathione peroxidase-4 (GPx4), **C** catalase (CAT), **D** malondialdehyde (MDA), **E** interleukin-1β (IL-1β), **F** solute carrier family 7 member 11 (SLC7A11), and **G** nuclear factor erythroid-2–related factor 2 (Nrf2) in the untreated CIM group (Group 2). Spearman correlation coefficients (*r*) demonstrated strong negative correlations between diarrhea score and antioxidant markers (SOD: *r* = − 0.716, GPx4: *r* = − 0.678, CAT: *r* = − 0.698, all *p* < 0.01) and strong positive correlations with oxidative/inflammation-related markers (MDA: *r* = 0.854, IL-1β: *r* = 0.831, *p* < 0.001). SLC7A11 and Nrf2 also showed significant negative correlations (*r* = − 0.891, *r* = − 0.724, respectively; *p* < 0.001 and *p* < 0.01). These correlations indicate that higher diarrhea severity is associated with increased oxidative/ferroptotic activity and reduced antioxidant defense

## Discussion

Chemotherapy-induced intestinal mucositis (CIM) is a frequent and clinically significant complication of anticancer therapy, manifesting as anorexia, abdominal discomfort, vomiting, and diarrhea, all of which contribute to malnutrition, increased susceptibility to infection, interruptions in cancer treatment, and even mortality (Chen et al. 2020).

There are no viable therapeutic approaches for the treatment of CIM, despite decades of experimental and clinical research (Ribeiro et al. 2016; Bowen et al. 2019). Thus, there is a pressing need to find new treatments for CIM.

**Fig. 9:**
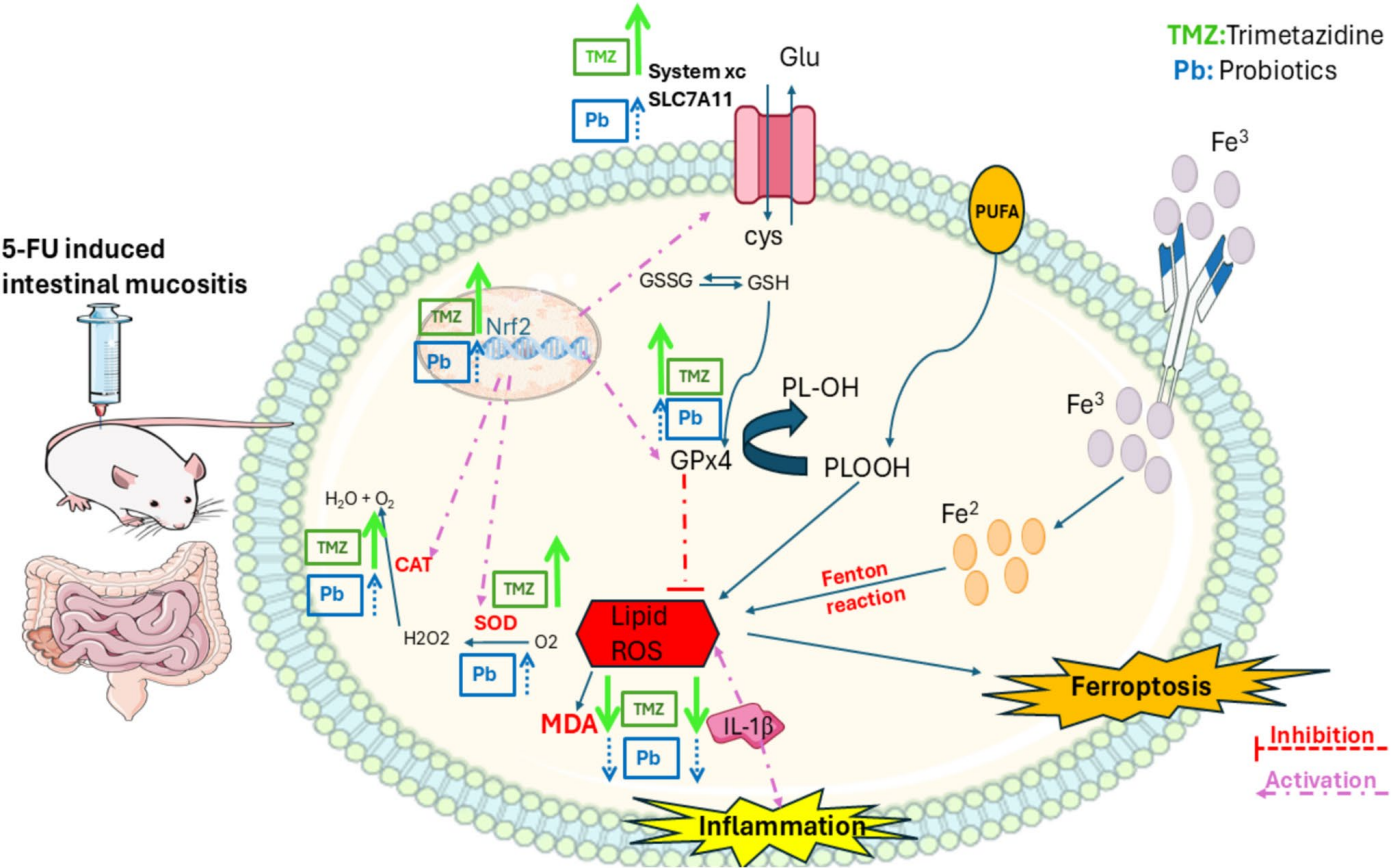
A mechanistic figure showing the mechanisms of trimetazidine and probiotics in attenuating 5-FU-induced intestinal mucositis; trimetazidine exerts a stronger activation of the Nrf2/SLC7A11/GPx4 axis, reflected by thicker green arrows. Probiotics also modulate oxidative and inflammatory markers but with comparatively milder effects, represented by thinner blue arrows

In the present work, the potential protective effects of trimetazidine (TMZ) and probiotics, individually and in combination, were investigated against 5-fluorouracil (5-FU) induced intestinal mucositis in mice, with a particular emphasis on oxidative stress, inflammation, and ferroptosis-related pathways involving the Nrf2/SLC7A11/GPx4 axis. The 5-FU mouse model was selected because it is rapidly developed, cost-effective, and closely reflects the pathological features observed in human CIM, making it a widely used approach for evaluating therapeutic candidates (Huang et al. 2022) (Fig. 9).

In the present study, following 5-FU administration, mice developed progressive body-weight loss and worsening diarrhea scores, findings fully consistent with earlier reports (Kissow et al. 2013; Pereira et al. 2016; Hamouda et al. 2017; Li et al. 2017; Yoneda et al. 2021). This might be explained as follows: the 5-FU injection considerably injures gastric epithelium in the stomach and small intestine tissue, displayed with subsequent malnutrition, malabsorption, and mucositis with diarrhea that led to progressive body-weight loss.

Histopathological evaluation further confirmed severe mucosal disruption characterized by villus shortening, crypt hypoplasia, epithelial necrosis, edema, and inflammatory cell infiltration. These alterations were accompanied by marked elevations in IL-1β, supporting the established role of inflammatory cytokines in the pathogenesis of mucositis (Guabiraba et al. 2014; Chen et al. 2016). In parallel, animals in the untreated CIM group demonstrated a clear state of oxidative stress, reflected by reduced SOD, CAT, and GPx4 and increased MDA levels, which agrees with prior 5-FU mucositis models in mice and rats (Rtibi et al. 2018; Ali et al. 2019; Yoneda et al. 2021)*.*

Accumulating evidence suggests that ferroptosis—a regulated, iron-dependent form of cell death characterized by phospholipid peroxidation—may contribute to chemotherapy-induced gastrointestinal injury. Classical features of ferroptosis include impaired antioxidant defenses, reductions in SLC7A11-mediated cystine uptake, diminished GPx4 activity, and the presence of redox-active iron (Bayır et al. 2020; Fang et al. 2020; Lin et al. 2020; Hong et al. 2021).

In the present study, the untreated CIM group exhibited markedly decreased SLC7A11 levels, reduced GPx4, and substantial iron deposition on Prussian blue staining. The coexistence of high tissue iron with suppressed SLC7A11 and GPx4 is an expected ferroptosis-related profile, suggesting that ferroptotic vulnerability may exacerbate mucosal injury. Notably, SLC7A11 has not previously been evaluated in CIM, indicating that the current study provides novel insight regarding ferroptosis-associated molecular alterations within CIM following 5-FU exposure.

Nrf2 is a central regulator of intracellular redox homeostasis and plays a key role in controlling ferroptosis-related defenses. Prior evidence confirms that Nrf2 transcriptionally regulates two major ferroptosis-protective targets—SLC7A11 and GPx4 (Dodson et al. 2019). Consistent with these mechanisms, our untreated CIM group demonstrated a marked reduction in Nrf2 levels compared with controls, supporting the association between impaired Nrf2 signaling and enhanced susceptibility to oxidative and ferroptotic injury.

Correlation analysis further supported the functional relevance of our findings. Diarrhea severity showed significant negative correlations with SOD, CAT, GPx4, SLC7A11, and Nrf2, alongside positive correlations with IL-1β and MDA. These patterns illustrate the interconnected contribution of oxidative stress, inflammation, and ferroptosis-related vulnerability to the clinical manifestations of CIM. Importantly, these correlations show co-occurrence rather than causation, indicating that the biochemical and clinical changes develop alongside each other but do not necessarily imply a direct cause-and-effect relationship.

TMZ demonstrated clear protective effects. Consistent with previous evidence indicating that TMZ may enhance Nrf2 activation and antioxidant enzyme expression (Zhao 2019), TMZ treatment in this study resulted in significant increases in SOD, CAT, GPx4, SLC7A11, and Nrf2, together with reductions in MDA and IL-1β. Histologically, TMZ markedly restored villus height, crypt depth, epithelial integrity, and enteritis score, with reduced iron deposition and improvements in body weight and diarrhea severity. These findings align with the known antioxidant and anti-inflammatory actions of TMZ reported in other experimental models (Eid et al. 2021), positioning TMZ as a promising candidate for CIM mitigation.

This study revealed that the probiotics-treated group provided antioxidant activity, which was proven by the significant increase in SOD, GPx4, and CAT and a significant decrease in MDA when compared to the untreated CIM group. In this context, the results were in harmony with Lee and Kang (2022), who proved that probiotics increased SOD, GPx4, and CAT tissue levels and reduced MDA levels in hepatocytes and liver tissues with induced oxidative stress.

Probiotics also exerted beneficial effects. The strains used—*Lactobacillus acidophilus* and *Bifidobacterium bifidum*—have previously been shown to strengthen epithelial barrier integrity, suppress pro-inflammatory cytokines, and promote redox homeostasis in intestinal injury models (Muñoz-quezada and Gil 2012; Kato et al. 2017; Al-sadi et al. 2021a, b; Al-sadi et al. 2021a, b). In agreement with these findings, the probiotics-treated group exhibited significant increases in SOD, CAT, and GPx4, along with reductions in MDA and IL-1β. Probiotics also increased Nrf2 expression and improved mucosal architecture. These findings align with previous evidence demonstrating that certain probiotic strains can suppress IL-1β production by reducing oxidative stress and promoting Nrf2-associated antioxidant responses, which supports the observed improvement in mucosal integrity following PB administration (Yun et al. 2020; Karaca et al. 2022).

Notably, to the authors’ knowledge, the effect of probiotics on SLC7A11 expression has not been previously investigated, making the observed increase in SLC7A11 a potentially novel contribution. Additionally, emerging evidence suggests that probiotics may influence ferroptosis-related pathways in certain inflammatory conditions, providing a biologically plausible context for our findings. In the present study, the antioxidant and anti-inflammatory effects observed with probiotic administration may help explain the reduction in lipid peroxidation and the improvement in mucosal status; however, these interpretations reflect associations and do not establish a direct mechanistic link (Li et al. 2022).

In this study, the effects of the combination of trimetazidine and probiotics were investigated. This combination group provided a significant increase in body weight, SOD, Gpx4, CAT, SLC7A11, Nrf2 levels, villi height, and crypt depths, and it also revealed a significant decrease in diarrhea score, MDA, and IL-1β levels as compared to the untreated CIM group. Moreover, the histopathological examination showed almost normal intestinal tissue.

The combination of trimetazidine and probiotics produced broad protective effects across inflammatory, oxidative stress, and ferroptosis-related markers. Notably, the combination therapy yielded clear improvements in GPx4 activity and intestinal structural recovery (villi height and crypt depth), demonstrating an additive benefit over either agent alone in these specific domains. However, it is important to acknowledge that this superiority was not consistent across all biochemical parameters: markers such as SOD, CAT, MDA, IL-1β, SLC7A11, and Nrf2 did not differ significantly between the combination and TMZ alone. Therefore, the combined regimen appears to provide a selective, rather than universal, enhancement of therapeutic effects, particularly strengthening outcomes related to antioxidant enzyme function and mucosal architecture.

On the other hand, when the combination group was compared to the probiotics-treated group, it provided superiority with regard to antioxidants, anti-inflammatory, and anti-ferroptosis biochemical markers represented by a significant increase in SOD, Gpx4, SLC7A11, and Nrf2 levels, and a significant decrease in MDA and IL-1β levels. However, it revealed a non-significant difference in CAT activity.

## Limitations

The present study has several limitations that should be acknowledged. First, functional inhibition or genetic knockdown models were not included; therefore, the involvement of the Nrf2/SLC7A11/GPx4 axis should be interpreted as associative rather than causative. Additionally, direct measurements of ferroptosis-related endpoints—such as labile Fe^2^⁺ quantification, GSH/GSSG ratios, and protein-level expression of key markers (e.g., Western blot for GPx4 or SLC7A11)—were not performed. Instead, ferroptosis-related alterations were inferred from biochemical changes (SLC7A11, GPx4, MDA, Nrf2) together with histological iron deposition. Iron accumulation was assessed using Prussian blue staining, which, while informative, is semi-quantitative and does not directly evaluate the labile iron pool, warranting incorporation of quantitative iron assays in future work. Furthermore, the study did not assess probiotic colonization or gut microbiota changes (e.g., 16S rRNA sequencing or fecal CFU counts); thus, microbial engraftment and microbiota-dependent mechanisms remain inferred rather than experimentally confirmed. Future studies integrating mechanistic inhibition, quantitative ferroptosis assays, and microbiota profiling will be essential to provide more definitive mechanistic insight.

## Conclusion 

The novel insight of this study is that trimetazidine and probiotics ameliorate 5-FU–induced intestinal mucositis through anti-inflammatory, antioxidant, and ferroptosis-related pathways. Trimetazidine exerted consistent protective effects across most biochemical and histological parameters, whereas the combination therapy offered selective additive benefits, particularly in intestinal structural recovery. These findings support the involvement of the Nrf2/SLC7A11/GPx4 axis in mediating the observed protection and highlight trimetazidine, alone or with probiotics, as promising candidates for mitigating chemotherapy-induced mucosal injury. Further studies are needed to clarify the mechanistic interactions between the two agents and to evaluate their behavior in advanced systems, including clinical models and cancer-bearing settings.

## Supplementary Information

Below is the link to the electronic supplementary material.

ESM 1 (DOCX 16.0 KB)

## Data Availability

The datasets generated and analyzed during the current study are available from the corresponding author on reasonable request.

## Abbreviations

*5-FU*: 5-Fluorouracil
*CIM*: Chemotherapeutics-induced intestinal mucositis
*ROS*: Reactive oxygen species
*IL*: Interleukins
*NF-κB*: Nuclear factor-kappa B
*SLC7A11*: Solute carrier family 7 member 11
*SLC3A2*: Solute carrier family 3 member 2
*GSH*: Reduced glutathione
*GPx4*: Glutathione peroxidase 4
*Nrf2*: Nuclear factor erythroid-related factor 2
*CAT*: Catalase
*SOD*: Superoxide dismutase
*MDA*: Malondialdehyde
*FAO*: Food and Agriculture Organization
*WHO*: World Health Organization
*DMSO*: Dimethyl sulfoxide
*i.p.*: Intraperitoneal injection
*TMZ*: Trimetazidine
*PB*: Probiotics
*B.W.*: Body weight
*H&E*: Hematoxylin and eosin
*PBS*: Phosphate-buffered saline
*ELISA*: Enzyme-linked immunosorbent assay
*SD*: Standard deviation
*IQR*: Interquartile range
*TNF-α*: Tumor necrosis factor-α
*TBHQ*: Tert-butylhydroquinone
