# Supplementary material for: Targeting ferroptosis by trimetazidine and probiotics to attenuate 5-fluorouracil-induced intestinal mucositis in mice
Source: Naunyn Schmiedebergs Arch Pharmacol. 2026 Jan 16;399(6):8959–73. doi: 10.1007/s00210-025-04936-9 (PMC13086825; doi:10.1007/s00210-025-04936-9)
Supplement: Supplementary file 1 — (DOCX 16.0 KB) [file 210_2025_4936_MOESM1_ESM.docx]

**Table (S-1): Difference between the initial and the final body weight (B.W) within each of the different studied groups.**

| ***Groups*** | ***Body Weight (B.W.)***  ***Mean ± SD***  ***n=10*** | |
| --- | --- | --- |
|  | ***Initial*** | ***Final*** |
| **Group 1**  **(Normal control group)** | 21.70± 2.62 | 27.40± 2.63 |
| **Group 2**  **(****Untreated group)** | 25.30 ± 2.21 | 21.50 ± 1.51 |
| **Group 3**  **(TMZ treated group)** | 25.20 ± 2.39 | 29.70± 2.49 |
| **Group 4**  **(****PB treated group)** | 24.30 ± 2.21 | 28.30 ± 2.21 |
| **Group 5**  **(TMZ + PB treated group)** | 25.10 ± 2.28 | 29.80± 1.75 |
